# Cephalometric Measurements of Upper Airway at Upright and Supine Posture in Adult OSAS Patients

**DOI:** 10.1055/s-0045-1811273

**Published:** 2025-09-04

**Authors:** Chun Hown Ng, Guang Chu, Min Gu, Yiu Yan Leung, Lizhuo Lin, Yanqi Yang

**Affiliations:** 1 Division of Paediatric Dentistry and Orthodontics, Faculty of Dentistry, The University of Hong Kong, Sai Ying Pun, Hong Kong SAR, People's Republic of China; 2 Division of Oral and Maxillofacial Surgery, Faculty of Dentistry, The University of Hong Kong, 34 Hospital Road, Sai Ying Pun, Hong Kong SAR, People's Republic of China

**Keywords:** obstructive sleep apnea, Chinese, supine, upright, posture

## Abstract

**Objective:**

This article aims to investigate whether posture (upright vs. supine) affects airway-related cephalometric measurements in adult Chinese patients with obstructive sleep apnea syndrome (OSAS).

**Materials and Methods:**

Twenty-seven adult Chinese male patients with OSAS (mean age, 50.1 ± 10.2 years), diagnosed through polysomnography, were recruited. All of the recruited patients had their lateral cephalograms taken in the upright posture and lateral cephalometric images taken using spiral computed tomography scans in the supine posture within a 3-month interval. Healthy Chinese men were included as the control group. Fourteen cephalometric parameters were measured using the images of the upper airway and craniofacial structures taken in the upright and supine postures.

**Statistical Analysis:**

A paired Student's *t* -test was applied to assess the differences between the two radiographic images, with statistical significance defined as *p* < 0.05.

**Results:**

Significant differences were observed in the distance between the tip of the soft palate and the posterior pharyngeal wall, tongue length, tongue height, and hyoid bone ( *p* < 0.05). These findings indicate that when the body posture was changed from upright to supine, the hyoid bone moved inferiorly and in the forward direction and the soft palate and tongue became thicker and shorter. No statistically significant difference was observed in the narrowest pharyngeal airway space between the upright and supine postures ( *p* > 0.05).

**Conclusion:**

Posture mainly affects the cephalometric measurements of the soft palate and tongue and the hyoid bone position. PASmin and U-MPW can still serve as reliable markers in upright lateral cephalometric radiographs for early detection of OSAS.

## Introduction

Obstructive sleep apnea syndrome (OSAS) is a sleep disorder that affects breathing during sleep and is characterized by repetitive interruption of ventilation during sleep caused by collapse of the pharyngeal airway. A diagnosis of OSAS is made when a patient has an apnea–hypopnea index (AHI; number of apnea and hypopnea events per hour of sleep) > 5 and symptoms of daytime sleepiness. 1 Habitual loud snoring, poor concentration, and fatigue are also common signs and symptoms. 2 Patients with untreated and undiagnosed OSAS report poorer quality of life due to excessive daytime sleepiness and are at risk of accidents. 3 OSAS has been associated with an increased risk of cardiovascular diseases, such as hypertension, heart failure, and stroke, and an elevated risk of early all-cause and cardiovascular mortality. 4 5 6

The prevalence of OSAS is 4.1% in Hong Kong 7 and 9 to 38% worldwide, as per a systematic review by Senaratna et al. 8 Sex differences in the prevalence of OSAS have also been reported, with a significantly higher prevalence in men than in women. 9

Early detection and screening of undiagnosed OSAS are crucial. Overnight polysomnography (PSG) remains the gold standard for diagnosing OSAS, but it requires a hospital setting, is expensive, and is not practical for rapid screening. Questionnaires such as STOP-Bang 10 and the Epworth sleepiness scale 11 have emerged as reliable, easy-to-use tools for early screening of OSAS, but their reported sensitivity levels vary widely. 12

Dentists or orthodontists routinely take lateral cephalometric radiographs (LCRs), especially for orthodontic diagnoses. As the image of the airway can be seen on LCRs, besides being useful in orthodontic diagnoses for the analysis of skeletal patterns, LCRs are proposed as a screening tool for OSAS, 13 and studies 14 15 have been conducted to compare lateral cephalometric parameters between individuals with and without OSAS.

However, as OSAS is a sleep disorder that is diagnosed with the patient in a supine posture, LCRs are taken in an upright posture, a clinical question that remains is whether the effect of posture needs to be taken into consideration when assessing the upper airway using LCRs. Previous studies have shown conflicting results in this regard. Pae et al 16 reported that the body posture has a substantial effect on upper airway structure and muscle activity. Yildirim et al 17 reported changes in the uvula width and hyoid bone movement with changes in posture. In contrast, Hsu and Wu 18 observed no statistically significant difference when examining the oropharyngeal airway and its relationship with changes in posture in healthy subjects without OSAS.

A very limited number of studies have focused on OSAS patients. In addition, the relevant literature has predominantly reported on patient samples from Caucasian populations. Thus, to the best of our knowledge, there is a dearth of research pertaining to the comparative assessment of airway dimensions in Chinese populations. This gap in the literature relates not only to studies involving individuals without OSAS but also extends to those investigating OSAS patients.

## Objectives

Therefore, the primary objective of this study was to compare the effect of posture on the airway structures of Chinese OSAS patients and to identify changes in their soft tissue and airway parameters in the supine posture relative to the upright posture. Notably, this study encompassed a broad range of parameters to assess the upper airway and associated structures. The sample was restricted to Chinese men to exclude the effects of sex and racial differences.

The secondary objectives of the study were to compare the soft tissue and airway parameters of OSAS patients with healthy controls to identify the parameters that are significantly different between these groups and can, thus, be used as a reference for the rapid screening of OSAS, and to identify those parameters that are affected by posture.

## Materials and Methods

This study was conducted in accordance with the ethical principles outlined in the Declaration of Helsinki. As a retrospective study, the research utilized preexisting clinical records and radiographic data, with no additional interventions imposed on patients. Ethical approval for this study was granted by the Institutional Review Board of the University of Hong Kong/Hospital Authority Hong Kong West Cluster (AU-21-519), which waived the requirement for individual informed consent. All imaging was processed to ensure complete de-identification prior to analysis and can only be accessed by authorized research personnel on encrypted devices, adhering to institutionally required data confidentiality protocols.

### Subjects

Southern Chinese individuals were recruited according to the following inclusion criteria: male, adult, OSAS confirmed with an overnight PSG report, and LCRs taken in the upright posture and lateral cephalometric images taken using spiral computed tomography (CT) scans in the supine posture within a 3-month interval. The exclusion criteria were patients with craniofacial syndrome, non-adult patients, and patients with a history of tonsillectomy or adenoidectomy. Twenty-eight male participants with a mean age of 50.1 ± 10.2 years were recruited for this study.

In addition, we conducted a comparative analysis with the normative data reported by Samman et al, 15 who conducted measurements of upper airway dimensions using LCRs in a healthy population of 29 Southern Chinese individuals.

### Sample Size Calculation

The sample size was calculated with reference to previous studies by Pae et al 16 and Yildirim et al, 17 based on an estimated intraclass correlation coefficient (ICC) > 0.8, to identify a significant agreement, with power set at 80% and the significance level set at 5% (two-sided). Accordingly, a sample of 24 was determined to be sufficient.

### Cephalometric Analysis

All measurements were performed by a single assessor (C.H.N.) after initial calibration on 10 LCRs and lateral cephalometric images from spiral CT with a 2-week washout interval. Another 10 randomly selected LCRs and lateral cephalometric images from spiral CT were measured by a second accessor (G.C.). The ICC was used to assess the inter-rater or intra-rater reliability.

LCRs were taken using a standardized technique in which the natural head position and the teeth were occluded. A Philips Orthoralix SD X-ray machine (Philips Medical Systems, Monza, Italy) and Kodak Ortho-G 15 × 30 film (Eastman Kodak Company, Rochester, New York, United States) were used. Using this machine, the distance from the mid-sagittal plane to the film was 15 cm, and the magnification ratio of the LCRs was calculated to be 12.5%.

Spiral CT scans were taken at the Prince Philip Dental Hospital using a standardized technique, with the patient awake in the supine posture. A GE MEDICAL SYSTEMS HiSpeed machine was used. The field of view of the spiral CT image extended from the base of the skull to the inferior border of the hyoid bone. The lateral cephalometric images were extracted from CT scans in the mid-sagittal plane by locating the ANS-PNS-IF (anterior nasal spine–posterior nasal spine–incisive foramen). 19 20 The validity and reliability of locating the mid-sagittal plane by both the primary (C.H.N.) and secondary (G.C.) assessors were tested with a 2-week wash-out interval.

### Landmarks and Measurements

The upper airway dimensions were measured from the LCRs and the lateral cephalometric images derived from spiral CT using landmarks and reference lines ( Tables 1 and 2 ). The LCRs were scanned using an HP ScanJet scanner and digitized and measured using CASSOS software (Soft Enable Technology Limited, Hong Kong SAR, People's Republic of China). The lateral cephalometric images from spiral CT scans were evaluated using Mimics software (version 20.0; Materialise, Leuven, Belgium).

**Table 1:** Cephalometric landmarks and measurements of the upper airway and craniofacial structures

	Abbreviation	Name	Description
Upper airway	PM-UPW U-MPW V-LPW PASmin	Nasopharyngeal airway space Oropharyngeal airway space Hypopharyngeal airway space Narrowest sagittal airway space in the pharynx	Distance from PM to UPW Distance from U to MPW Distance from V to LPW The shortest distance between point Z and PPW, where point Z is not a fixed landmark
Soft palate	SPT PM-U NL/PM-U	Soft palate thickness Soft palate length Soft palate angle	Maximum thickness of the soft palate perpendicular to PM-U Distance from PM to U Angle between PM-U and NL
Tongue	TH TL	Tongue height Tongue length	Distance from H to VT Distance from V to T
Hyoid bone	AH-MP AH-FH AH-Me AH-Rgn AH-Cv	Vertical position of the hyoid bone with respect to the mandible Vertical position of the hyoid bone with respect to the Frankfort plane Horizontal position of the hyoid bone with respect to the menton Horizontal position of the hyoid bone with respect to the retrognathion Horizontal position of the hyoid bone with respect to the cervical vertebra	Distance from AH to MP Distance from AH to FH Distance from AH to Me Distance from AH to Rgn Distance from AH to Cv

**Table 2:** Cephalometric landmarks and reference lines for airway evaluation

Abbreviation	Name	Description
AH	Anterior hyoid	The most anterior and superior point on the body of the hyoid
Cv	Cervical vertebra	Line joining the second and third cervical vertebra
FH	Frankfort horizontal plane	Line joining Or to Po
U	Uvula	Tip of uvula
UPW	Upper pharyngeal wall	Point of intersection of the line perpendicular to the posterior pharyngeal wall from the posterior nasal spine
MPW	Middle pharyngeal wall	Intersection of the perpendicular line from U to the posterior pharyngeal wall
LPW	Lower pharyngeal wall	Intersection of the perpendicular line from V to the posterior pharyngeal wall
PM	Pterygomaxillary	The point at the junction of the pterygomaxillary and the posterior nasal spine
V	Vallecula	Intersection of the epiglottis and the base of the tongue
NL	Nasal line	The line between the anterior nasal spine (ANS) and the posterior nasal spine (PNS)
T	Tip of the tongue	The most anterior point of the tip of the tongue
H	Superior part of the tongue	The most superior point of the tongue in relation to the line VT
MP	Mandibular plane	Line joining Me to Go'
Me	Menton	The most inferior point of the symphysis
Rgn	Retrognathion	The most posterior point of the mandible symphysis

**Fig. 1:**
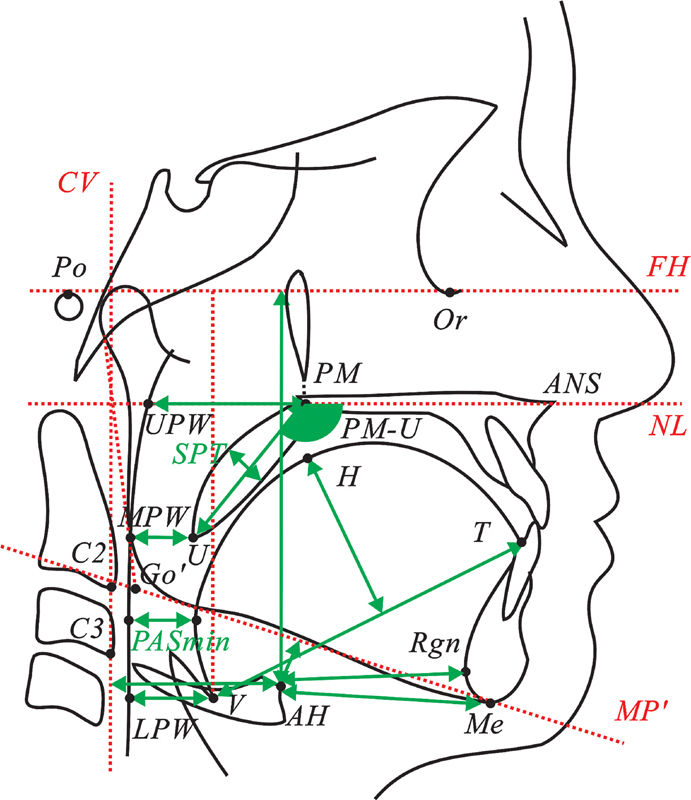
Cephalometric landmarks and measurements of the upper airway and craniofacial structures.

**Fig. 2:**
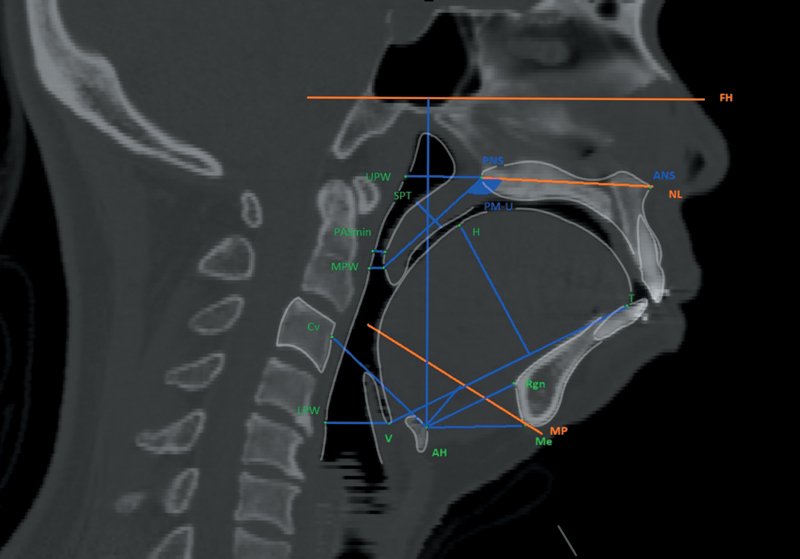
Lateral cephalometric images from spiral CT scans in the mid-sagittal plane and corresponding measurements.

The reference points and lines used were taken and modified from Samman et al 15 and Savoldi et al. 21 These reference points and lines have also been widely used in the literature. 13 16 17 22 Detailed descriptions of the parameters measured and their abbreviations are shown in Tables 1 and 2 . Fig. 1 illustrates the cephalometric landmarks and measurements identified on lateral cephalograms; Fig. 2 presents the corresponding landmarks and measurements extracted from CT images. Fourteen parameters, including 13 linear distances and 1 angle, were identified and measured.

The upper airway space was measured in the sagittal plane at four levels: the nasopharynx (PM-UPW), oropharynx (U-MPW), hypopharynx (V-LPW), and narrowest pharyngeal airway space (PASmin). The thickness (SPT), length (PM-U), and angle (NL/PM-U) between the long axis (PM-U) and the nasal line (NL) of the soft palate were measured. The tongue was represented by its length (TL) and height (TH). The hyoid bone position was measured in both the sagittal and vertical planes.

### Statistical Analysis

Statistical analysis was performed using SPSS Statistics 20 (IBM, Armonk, New York, United States). A paired Student's *t* -test was used to evaluate the differences between two radiographic images. Statistical significance was set at *p* < 0.05.

## Results

From the initial pool of 36 OSAS patients, eight were excluded due to incomplete records, and one was excluded due to the different types of X-ray machines used to obtain the LCRs. Thus, the final sample of this study included 27 OSAS patients.

### Assessment of Reliability

A comparison of the two sets of measurements (T1 vs. T1′) taken 2 weeks apart by the primary assessor (C.H.N.) was performed to assess the intra-rater reliability. The overall ICC values ranged from 0.825 to 0.995. The ICC for intra-rater agreement in the mid-sagittal plane coordinates was 0.973, indicating excellent reliability.

### Effect of Posture (Upright and Supine) on the Upper Airway

Table 3 shows a comparison of measurements between supine and upright postures in the OSAS group. The PASmin was slightly reduced in the supine posture relative to the upright posture, but the difference was not statistically significant ( *p* = 0.078).

**Table 3:** Cephalometric measurements of OSAS patients in the supine and upright postures

Parameters	Supine (mm)	Upright (mm)	*p* -Value
Soft palate			
PM-U (soft palate length)	36.0	36.8	0.213
SPT (soft palate thickness)	10.7	9.6	0.003 a
NL/PM-U (angle between soft palate and maxillary plane)	130	128.7	0.36
Upper airway dimension			
PM-UPW (PNS to posterior wall)	21.9	21.6	0.769
U-MPW (tip of soft palate to posterior wall)	6.3	8.8	0.023 a
V-LPW (epiglottis to posterior wall)	17.8	17.6	0.781
PASmin	4.4	4.9	0.078
Tongue			
TL (tongue length)	70.7	74.8	<0.001 a
TH (tongue height)	38.4	36.9	0.028 a
Hyoid bone			
H-MP (hyoid to mandibular plane)	22.5	21.7	0.472
H-FH (hyoid to Frankfurt plane)	106.1	99.1	<0.001 a
H-Me (hyoid to menton)	35.7	38.0	0.023 a
H-Rgn (hyoid to retrognathion)	36.6	36.7	0.905
H-CV (hyoid to cervical bone)	41.7	35.7	<0.001 a

a *p* -Value<0.05.

#### Soft Palate

In the supine posture, the soft palate showed a significant increase in thickness by 1.1 mm ( *p* = 0.003) and a nonsignificant decrease in length by 0.8 mm ( *p* = 0.213) relative to the upright posture.

#### Upper Airway

No significant differences were observed in the PM-UPW and V-LPW between the two postures. However, the U-MPW was reduced from 8.8 mm in the upright posture to 6.3 mm in the supine posture, and this difference was statistically significant ( *p* = 0.023).

#### Tongue

Both the TL and TH showed significant differences with changes in posture. The TL decreased from 74.8 mm in the upright posture to 70.7 mm in the supine posture, whereas the TH increased from 36.9 mm in the upright posture to 38.4 mm in the supine posture.

#### Hyoid Bone

The hyoid bone was assessed in vertical and sagittal dimensions. In the vertical dimension, the H-MP and H-FH increased by 0.8 and 7 mm, respectively, in the supine posture relative to the upright posture. In the sagittal dimension, the H-Me decreased by 2.3 mm, while the H-CV increased by 6 mm in the supine posture relative to the upright posture. The hyoid bone moved inferiorly and in the forward direction in the supine posture relative to the upright posture.

### Comparison of Cephalometric Measurements between OSAS Patients and Healthy Controls

Table 4 shows a comparison of cephalometric measurements between OSAS patients and healthy controls.

**Table 4:** Comparison of cephalometric measurements taken in the upright and supine postures in OSAS patients with normal measurements taken in healthy controls

Parameters	OSA (upright)			OSA (supine)			Posture related (Y/N)
	Norm (Ref) (mm)	Measurements (mm)	*p* -Value	Norm (Ref) (mm)	Measurements (mm)	*p* -Value	
PM-U (soft palate length)	34.3	36.8	0.028 a	34.3	36	0.1006	N ( *p* = 213)
SPT (soft palate thickness)	10.1	9.6	0.187	10.1	10.7	0.1679	Y ( *p* = 0.003 a )
NL/PM-U (angle between soft palate and maxillary plane)	127.4	128.7	0.41	127.4	130	0.0996	N ( *p* = 0.36)
PM-UPW (PNS to posterior wall)	25.9	21.6	<0.001 a	25.9	21.9	<0.001 a	N ( *p* = 0.769)
U-MPW (tip of soft palate to posterior wall)	9.9	8.8	0.313	9.9	6.3	<0.001 a	Y ( *p* = 0.023 a )
V-LPW (epiglottis to posterior wall)	18.7	17.6	0.257	18.7	17.8	0.26	N ( *p* = 0.781)
PASmin	10.1	4.9	<0.001 a	10.1	4.4	<0.001 a	N ( *p* = 0.078)
TL (tongue length)	72	74.8	0.045 a	72	70.7	0.3017	Y ( *p* <0.001 a )
TH (tongue height)	36.9	36.9	1	36.9	38.4	0.0997	Y ( *p* = 0.028 a )
H-MP (hyoid to mandibular plane)	NA	21.7	NA	NA	22.5	NA	N ( *p* = 0.472)
H-FH (hyoid to Frankfurt plane)	92.4	99.1	<0.001 a	92.4	106.1	<0.001 a	Y ( *p* <0.001 a )
H-Me (hyoid to menton)	NA	38	NA	NA	35.7	NA	Y ( *p* = 0.023 a )
H-Rgn (hyoid to retrognathion)	NA	36.7	NA	NA	36.6	NA	N ( *p* = 0.905)
H-CV (hyoid to cervical bone)	36.4	35.7	0.593	36.4	41.7	0.003 a	Y ( *p* <0.001 a )

a *p* -Value<0.05.

Among the included cephalometric measurements, the PASmin showed the greatest differences between OSAS patients and healthy controls. The mean PASmin in the OSAS group was 4.9 mm relative to 10.1 mm in the control group. However, the PASmin showed no statistically significant difference with changes in posture.

The soft palate length and PM-UPW were significantly increased in OSAS patients compared with healthy controls, and the TL and H-FH were also significantly increased in OSAS patients, indicating a longer tongue and lower position of the hyoid bone in the patients. The soft palate thickness and U-MPW were affected by changes in posture. The TH, TL, and hyoid bone measurements also showed differences between the two postures.

## Discussion

As mentioned in the “Introduction,” LCRs can provide information related to the airway. However, LCRs are taken in the upright posture, while OSAS problems caused by reduced airway dimensions may be more obvious in the supine posture. Therefore, it is worthwhile to understand whether the body posture affects the airway dimensions in cephalometric analysis. Previous studies have reported conflicting results in this regard, 16 17 22 with most of them being conducted in general populations without OSAS. 18 23 24 In addition, the few studies involving OSAS patients had small sample sizes. 23 Thus, the present study is the first to investigate the effects of posture on airway dimensions in Chinese OSAS patients. Furthermore, racial 25 differences in the prevalence of OSAS and nasal respiratory resistance (NRR) 26 have been reported. Thus, findings from Chinese OSAS patients offer dual value: they deliver locally relevant clinical benchmarks while providing the international research community with comparative data to inform future translational studies.

As OSAS is more common in men than in women 9 due to sex differences in normal airway dimensions, 15 our study included only Chinese men with OSAS to make the sample more representative and to enable reliable comparisons with the healthy male population reported in a previous study. 15

In addition to comparing the cephalometric measurements between supine and upright postures, this is the first study to investigate the key cephalometric parameters that can differentiate OSAS patients from individuals without OSAS and are affected by posture. Our results provide meaningful information for clinicians to interpret airway-related cephalometric parameters when using LCRs for identifying cases with potential airway problems.

### Effect of Changing the Posture from Upright to Supine on Airway Dimensions in OSAS Patients

#### Soft Palate

In the supine posture, the soft palate showed a statistically significant increase in thickness by 1.1 mm ( *p* = 0.003) and a nonsignificant decrease in length by 0.8 mm ( *p* = 0.213), relative to the upright position. This finding indicates that the soft palate tends to be thicker in the supine posture. These results are consistent with those reported by Pracharktam et al, 27 but conflict with those reported by Pae et al 16 and Yildirim et al, 17 who found that the soft palate length increases in the supine posture. This discrepancy may be explained by the relatively small sample size of 10 OSAS patients in the study conducted by Pracharktam et al 27 and the unclear visibility of the soft palate in LCRs.

In our study, we not only confirmed significant differences in soft palate dimensions between OSAS patients and healthy adults but also found that these dimensions are influenced by changes in posture. Additionally, we observed the U-MPW, which indicates the distance between the tip of the soft palate and the posterior wall, was significantly reduced by 2.5 mm in the supine posture compared with the upright posture ( *p* = 0.023), and this finding is consistent with those of previous studies. 16 17 This result may be explained by the alterations in soft palate dimensions and the effect of gravitational pull exerted on the soft palate and uvula. Consequently, in the supine position, the soft palate falls back posteriorly, which may narrow down the nasopharyngeal and oropharyngeal airways.

Previous studies by Habumugisha et al 28 using MRI to evaluate the soft palate and upper airway volume demonstrated a negative correlation between soft palate length and upper airway dimensions, indicating that patients with longer soft palates tend to have narrower upper airways. These findings are consistent with our results, further supporting the notion that soft palate morphology is closely associated with airway patency. This correlation highlights the potential value of soft palate assessment in the early identification of individuals at risk for OSAS, particularly during routine orthodontic evaluations.

#### Tongue

Our soft palate findings demonstrated that the tongue became shorter and thicker in the supine posture relative to the upright posture. These results are in agreement with those reported by Pae et al 16 and Ingman et al, 22 who observed similar trends of tongue deformation from the upright to supine postures. It is important to note that the tongue is not very clear in LCRs due to superimpositions with other structures (e.g., the mandible); therefore, Savoldi et al 21 stated that LCRs have limited reliability in assessing the tongue and soft palate, which may compromise the diagnostic application of LCRs.

#### Hyoid Bone

The hyoid bone was assessed using different parameters and reference planes in vertical and sagittal dimensions. In the vertical dimension, the H-MP and H-FH were measured. From the upright to supine posture, the MP-H showed a small, nonsignificant increase of 0.8 mm ( *p* = 0.472), while the H-FH showed a significant increase of 7 mm ( *p* < 0.001). This result indicates that the hyoid bone moved inferiorly in the supine posture.

In the sagittal dimension, the H-Me, H-Rgn, and H-CV were measured. Compared with the upright posture, our results revealed a significantly shorter distance between the hyoid bone and the menton, but a significantly longer distance between the hyoid bone and the cervical vertebra, in the supine posture, indicating that the hyoid bone moved forward in the supine posture.

In summary, the hyoid bone moved inferiorly and anteriorly when changing from the upright posture to the supine posture, and this result is in agreement with those of previous reports. 16 17 29 Jo et al 29 also suggested that the distance between the hyoid bone and mandibular plane can be a valuable diagnostic parameter for detecting patients with severe OSAS, with a longer MP-H distance indicating a higher AHI value. It has also been suggested that, as the hyoid bone moves to a lower position, it increases the pharyngeal length (distance between the posterior nasal spine and the base of the epiglottis, which is attached to the hyoid bone), and a longer pharyngeal length is associated with a higher risk of pharyngeal collapse and a higher AHI value. 30 31

It is important to note that the hyoid bone is anatomically connected to the tongue muscles, and its position is influenced by dynamic factors such as respiration, swallowing, and tongue movements. Clinically, it remains challenging to accurately define or quantify what constitutes an “inferior” position of the hyoid bone.

### Key Cephalometric Parameters that Differentiate OSAS Patients from Healthy Controls and Are Affected by Posture

To determine the key cephalometric parameters that differentiate OSAS patients from individuals without OSAS and are also affected by posture, we compared the cephalometric parameters of our OSAS patients obtained in supine and upright postures with normal measurements taken in healthy controls reported in a previous study. 16 As the normal parameters in the healthy population were established using LCRs, they were all measured in the upright posture. 16

For the soft palate, compared with measurements in the healthy population, the PM-UPW, U-MPW, and hypopharynx (V-LPW) were reduced in the sagittal dimension in OSAS patients, with the nasopharynx being significantly shorter in the latter. These results are in agreement with those of previous studies. 16 17 27 This finding confirmed that OSAS patients have an abnormal pharyngeal structure and function, especially in cases of severe OSAS.

Among the cephalometric parameters for the soft palate, only the U-MPW, which indicates the distance between the tip of the soft palate and the posterior wall, was significantly affected by posture. Specifically, it was significantly reduced in the supine posture relative to the upright posture. Even in healthy subjects, previous studies 16 17 have revealed a reduction in the U-MPW with a change in posture from upright to supine. Our results revealed that there was no significant difference between the U-MPW of OSAS patients in the upright posture and the corresponding measurement in the healthy population, but the U-MPW in the supine position was significantly shorter in our patient population than in the healthy population. This finding highlights that the U-MPW distance in LCRs, which are commonly taken in the upright posture, is overestimated, and with a change in posture to supine, it reduces significantly, indicating that it may render an even narrower oropharyngeal airway in OSAS patients.

The OSAS group had a significantly increased TL, but the tongue thickness remained the same, and both parameters were affected by postural changes. The genioglossus muscle, which is the main airway dilator muscle, is more hypotonic in OSAS patients than in individuals without OSAS. 32 Pae et al 16 suggested that this muscle plays an important role in the upper airway. Upper airway dimensions may be more affected by gravity in OSAS patients due to genioglossus hypotonicity or a heavier tongue. However, it is important to note that the image of the tongue is not very clear in LCRs due to superimposition with the body of the ramus and the mandibular teeth.

Only two hyoid bone parameters, namely, the H-FH and H-CV, could be compared, as no other parameters have been reported in the healthy Chinese population. The H-FH (distance between the hyoid bone and the Frankfort horizontal line) was significantly different in our patients relative to the healthy population, indicating that the hyoid bone moved inferiorly in the supine posture. This finding is consistent with the finding reported by Maschtakow et al, 33 who found that the H-MP was significantly increased by 25% in 26 Brazilian patients with OSAS relative to 29 healthy controls. Wong et al 34 conducted an interethnic comparative study on craniofacial morphology. They observed that in the Chinese moderate-severe group, the hyoid bone was located more caudally, and this measurement may be more diagnostically relevant for Chinese with OSAS. Weakened efficiency of hyoid muscles, including the genioglossus, may be a crucial factor contributing to pharyngeal obstruction.

Most importantly, the H-FH was significantly influenced by changes in posture, with the hyoid bone moving even more caudally in the supine posture. This finding highlights that the vertical position of the hyoid bone in upright LCRs may lead to an underestimation of its actual position in OSAS patients, meaning that the hyoid bone in the supine posture may be more inferior. It has been emphasized that the movement of the hyoid bone to a lower position increases the pharyngeal length (distance between the posterior nasal spine and the base of the epiglottis, which is attached to the hyoid bone), and a longer pharyngeal length is associated with a higher risk of pharyngeal collapse and a higher AHI value. 30 31

Not surprisingly, the PASmin showed the greatest difference between OSAS patients and the healthy population. The posterior airway was approximately twice as wide in the healthy group as in the OSAS group. Battagel and L'Estrange 35 concluded that the oropharynx dimensions are markedly reduced in OSAS patients; hence, LCRs may be of value for identifying individuals with OSA. In our study, the PASmin was not affected by posture, even though it showed a small, nonsignificant decrease in the supine posture. The insignificant variation in PASmin measurements across different postures substantiates its status as both a highly valuable diagnostic parameter for OSAS and a remarkably stable measure unaffected by positional changes. This stability renders PASmin a particularly reliable indicator when assessing OSAS risk through upper airway analysis using LCRs obtained in the upright position.

### Limitations and Perspectives of Future Studies

According to the report by Bixler et al, 36 the prevalence of obstructive sleep apnea (OSA) is significantly higher in males than in females, with a male-to-female patient ratio of 3.3:1. Besides, there is gender differences in normal airway dimensions. 15 Therefore, to eliminate interference from gender and ethnic differences, this study included only Chinese male participants. Although the sample size enrolled in this study was sufficient to detect meaningful effects, the interpretation of the results is mainly applicable to males, which should be cautiously generalized to different genders and ethnicities. Future research could incorporate gender as one of the investigative factors to explore its association with posture-induced changes in cephalometric parameters.

Ideally, there is value in comparing LCRs taken in the two different postures, but this is not feasible, as LCRs for dental and orthodontic examination are generally taken only in the upright posture. Therefore, we included individuals who had CT scans taken in the supine posture within a 3-month interval of their LCRs and then extracted lateral cephalometric images from their CT scans for comparison. As the lateral cephalometric images were derived from CT scans taken in the mid-sagittal plane, 19 20 37 and the reliability of locating the mid-sagittal plane was satisfactory (ICC = 0.973 and 0.936 for intra- and inter-rater agreement, respectively), the images were considered comparable to LCRs.

Soft tissues, such as the soft palate and tongue, were not clearly visible in the LCRs. Due to the retrospective nature of this study, coating the tongue to increase its visibility in the images was not possible. 21

As the patients were awake when undergoing the CT scans, the images may not reflect the actual situation when they are asleep. The suppression of pharyngeal muscle activity during sleep is critical to OSAS, as it renders a narrower upper airway that is more vulnerable to collapse. 38 A significant increase in pharyngeal collapsibility has been reported in sleep relative to wakefulness in both healthy controls and OSAS subjects. 39

This study did not account for variations in muscular tone between wakefulness and sleep states, instead focusing specifically on elucidating the postural effects on cephalometric outcomes. For orthodontic practitioners utilizing LCRs for airway assessment, these findings serve as an important clinical consideration: airway compromise may be underestimated in upright-posture radiographs. Consequently, where sleep-related exacerbation of airway obstruction is suspected, prompt referral to sleep specialists for PSG evaluation is recommended.

Since it is difficult to obtain both CT scan and lateral cephalogram taken at the same time, to minimize the likelihood of anatomical or physiological changes, a time interval of no more than 3 months was set between the CT scan and the lateral cephalogram. This time interval was smaller than some CBCT-related studies 1 2 on the effects of tooth extraction and nonextraction orthodontic treatment on airway structure which showed no significant changes in the three-dimensional structure of the upper airway with time intervals up to 24 months between CBCT acquisitions. Therefore, the likelihood of airway structural changes within a 3-month time interval is expected to be low with minimal effects on our current results. Nevertheless, it is ideal to have both images taken at the same points.

Due to the limitations of the retrospective study design as well as the unethical and impractical problem with obtaining CBCT data from matched healthy controls, this study employed an alternative comparative approach utilizing normative data from Samman et al. But such comparisons still have certain limitations. Future research would benefit from more rigorously designed prospective studies incorporating both healthy and OSAS cohorts with stringent control of confounding variables, to systematically elucidate potential physiological differences between these groups.

This study provides orthodontic clinicians with a valuable reference for identifying patients at high risk of OSAS. Orthodontists play a pivotal role in OSAS detection, as existing literature demonstrates a significantly higher prevalence of OSA among orthodontic patients, 40 41 with established correlations between OSAS and specific malocclusion-related craniofacial characteristics. 42

In routine orthodontic practice, concerns regarding cost and radiation exposure limit the universal application of CBCT imaging. Consequently, LCRs emerge as a practical screening modality for OSAS risk assessment. Our investigation analyzed cephalometric data obtained from OSAS patients in both upright and supine postures, elucidating the postural influences on upper airway parameters while validating PASmin as a robust diagnostic indicator.

These findings offer clinical reference to orthodontic practitioners, facilitating appropriate referral of patients with high risk of OSAS to sleep specialists or otorhinolaryngologists for comprehensive evaluation, as far as possible to avoid the negative consequences of delayed diagnosis and treatment.

## Conclusion

The change in posture from upright to supine mainly affects the soft palate, tongue, and hyoid bone position. The distance between the tip of the soft palate and the posterior pharyngeal wall was reduced, and the hyoid bone moved forward and inferiorly with a change in posture from upright to supine.

The caudal position of the hyoid bone was underestimated in LCRs taken in the upright posture, and the bone was more inferiorly placed in the supine posture relative to the upright posture in OSAS patients.

The oropharyngeal airway (U-MPW) was overestimated in LCRs taken in the upright posture, while it showed significantly reduced dimensions in the supine posture relative to the upright posture in OSAS patients.

The PASmin was significantly reduced in OSAS patients, but showed no significant difference with changes in posture.

PASmin and U-MPW can still serve as reliable markers in upright LCRs for early detection of OSAS.
